# Comparing outcomes of one-way Heimlich valve with conventional chest tube drainage for primary spontaneous pneumothorax: a randomized clinical trial

**DOI:** 10.1016/j.sipas.2025.100314

**Published:** 2025-10-13

**Authors:** Mohammad Hadi Bahri, Seyed Mohammad Naghibalghora, Mojtaba Ahmadinejad, Kourosh Kabir, Nazanin Khezri

**Affiliations:** a Assistant Professor, Department of Surgery, Shahid Madani Hospital, School of Medicine, Alborz University of Medical Sciences, Karaj, Iran; b Professor, Department of Surgery, Shahid Madani Hospital, School of Medicine, Alborz University of Medical Sciences, Karaj, Iran; c Associate Professor, Preventive Medicine and Public Health Research Center, Department of Community and Family Medicine, School of Medicine, Iran University of Medical Sciences, Tehran, Iran; d Associate Professor, Firoozabadi Clinical Research Development Unit, Department of Community and Family Medicine, School of Medicine, Iran University of Medical Sciences, Tehran, Iran; e General Practitioner(GP), Student Research Committee, Faculty of Medicine, Alborz University of Medical Sciences, Karaj, Iran

**Keywords:** One-way Heimlich Valve, Chest tube, Primary spontaneous pneumothorax

## Abstract

**Background:**

Primary spontaneous pneumothorax (PSP) is the accumulation of air in the pleural space without underlying lung disease. Standard management often involves chest tube insertion connected to an underwater seal drainage system (bottle), but alternatives like the Heimlich one-way valve exist. This study aimed to compare the clinical outcomes of using a Heimlich valve versus standard chest tube drainage for PSP.

**Methods:**

This was a single-center, open-label, parallel-group randomized controlled trial conducted at Shahid Madani Hospital, Karaj, Iran, from March 2023 to March 2024. Forty patients aged 18–40 years with symptomatic PSP (>15% collapse) were randomized (1:1 ratio) using block randomization. The intervention group received a 28 Fr chest tube connected to a Heimlich valve. The control group received a 28 Fr chest tube attached to an underwater sealed bottle. Primary outcomes included length of hospital stay, time to return to normal activities other outcomes included pain scores (Visual Analog Scale - VAS), dyspnea score (0–10), ease of getting out of bed (0–10), need for ketorolac analgesia, treatment failure (requiring VATS within 7 days), 30-day rehospitalization, and complications.

**Results:**

Forty patients (mean age 31.1±7.0 years; 80% male) were randomized to separate groups (20 per group). Baseline characteristics were similar between groups. The mean time to return to normal activities was significantly shorter in the Heimlich group (7.1±5.4 days vs. 10.2±8.0 days, P=0.014). Mean length of hospital stay was 5.6±3.0 days (Heimlich) vs. 7.3±4.6 days (Bottle), (P=0.081). Pain scores were significantly lower in the Heimlich group on days 1–4 (P<0.01). Ketorolac use (frequency and total dose) was significantly lower in the Heimlich group (P<0.001). Ease of getting out of bed was significantly greater in the Heimlich group throughout the assessment period. Pneumothorax resolution trended faster in the Heimlich group (P=0.077 on day 4). Dyspnea trended lower in the Heimlich group on day 4 (P=0.078). Treatment failure (requiring VATS) occurred in 1 (5%) of the Heimlich patients versus 3 (15%) of the Bottle patients (P = 0.29). Rehospitalization occurred in one patient per group (5%, P = 1.00).

**Conclusion:**

In patients with PSP, management with a Heimlich valve resulted in a significantly faster return to normal activities, lower pain scores, reduced analgesic requirements, and greater ease of mobilization than standard chest tube drainage. While not statistically significant, trends suggested faster pneumothorax resolution and potentially shorter hospital stays. The Heimlich valve appears to be a safe and effective alternative, offering potential patient comfort and recovery benefits.

**Trial registration:**

Iranian Registry of Clinical Trials (IRCT): IRCT20230208057359N1.

## Introduction

Primary spontaneous pneumothorax (PSP) is characterized by the abnormal presence of air in the pleural space in individuals who do not exhibit visible lung disease [1,2]. It generally results from the rupture of subpleural blebs or bullae, which are predominantly found in the apical region [3]. Although frequently viewed as benign, PSP poses a notable public health concern, especially among young, tall, slender males, who often have a smoking history [4,5].

The clinical presentation can differ, but typically features sudden pleuritic chest pain and shortness of breath [6,7]. Diagnosis is based on clinical suspicion validated by imaging, usually through chest radiography or, more frequently, point-of-care ultrasound [1,2]. Computed tomography (CT) provides high sensitivity and specificity but is generally reserved for ambiguous cases or surgical preparations [3].

Management aims to evacuate pleural air, facilitate lung re-expansion, and prevent recurrence [1]. Options range from observation for small, minimally symptomatic cases to interventional approaches like simple aspiration, chest tube insertion with underwater seal drainage (bottle), or surgical intervention, e.g., Video-Assisted Thoracoscopic Surgery (VATS) for recurrent or persistent cases [[2], [3], [4]]. Standard chest tube drainage connected to a bottle system is adequate but could limit patient mobility, potentially prolong hospital stay, and requires careful monitoring [5,6].

One alternative is using a one-way valve connected to the chest tube, such as the Heimlich valve [7]. This device allows air to exit the pleural space during expiration but prevents re-entry during inspiration, potentially facilitating ambulatory management, reducing hospital stay, and improving patient comfort [8]. However, comparative data, particularly from randomized trials focusing specifically on the Heimlich valve versus standard bottle drainage for the initial management of PSP, are still evolving [9,10]. While some studies have compared ambulatory approaches or pigtail catheters with Heimlich valves to chest tubes [11,12], direct comparisons using standard bore chest tubes in both arms but varying the drainage system (Heimlich vs. bottle) are less common.

This study aims to compare the clinical outcomes, including duration of hospital stay, pain levels, recovery time, and complication rates, associated with using a Heimlich one-way valve versus conventional chest tube drainage connected to an underwater sealed bottle in the initial management of patients with PSP.

## Methods

### Study design and setting

This study was a single-center, open-label, parallel-group randomized controlled trial conducted at Shahid Madani Hospital, Karaj, Iran, from March 2023 to March 2024. The Ethics Committee of Alborz University of Medical Sciences approved the study protocol, which was registered with the Iranian Registry of Clinical Trials (IRCT20230208057359N1).

### Participants

Eligible participants included patients aged 18 to 40 presenting with their first symptomatic primary spontaneous pneumothorax at Shahid Madani Hospital. The age cap of 40 was selected to align with the typical epidemiology of PSP, mainly affecting younger adults without pre-existing lung conditions. Patients older than 40 are more prone to secondary pneumothorax causes or age-related comorbidities like COPD, which could influence the study results [13]. Patients were required to be hemodynamically stable and provide informed consent.

Exclusion criteria included a history of thoracic trauma within the preceding month, severe asthma or Chronic Obstructive Pulmonary Disease (COPD), a history of COVID-19 within the prior month, known psychosis, the need for Intensive Care Unit (ICU) admission, complete lung collapse potentially indicating tension physiology or a high likelihood of persistent air leak, pregnancy or lactation, contraindications to chest tube insertion, conditions in which instantly requiring mandatory VATS (e.g., single lung, high-risk occupations like diver or pilot, large bullae identified initially), or refusal to participate.

### Randomization and allocation concealment

Patients meeting eligibility criteria were randomized in a 1:1 ratio to either the Heimlich valve group or the chest tube with bottle group. Randomization was performed using a block randomization method with blocks of four. The randomization sequence was generated using a computer program. Allocation concealment was maintained using sequentially numbered, opaque, sealed envelopes prepared by an independent research assistant not involved in patient recruitment or assessment. The envelope corresponding to the enrolled patient's sequence number was opened only after eligibility was confirmed and consent obtained.

### Blinding

Due to the nature of the interventions (visible external drainage system), blinding patients and treating clinicians/researchers assessing outcomes was not feasible. The study was conducted in an open-label manner.

### Interventions

All patients underwent chest tube insertion under local anesthesia using a standardized technique. A 28 French (Fr) chest tube was inserted in the 4th or 5th intercostal space, typically between the anterior and mid-axillary line, through a small (approx. 2 cm) incision. The position was confirmed radiographically.

#### Intervention group (Heimlich valve)

The distal end of the 28 Fr chest tube was connected to a sterile, commercially available Heimlich one-way valve. Patients were encouraged to mobilize as tolerated.

#### Control group (Bottle drainage)

The distal end of the 28 Fr chest tube was connected via tubing to a standard underwater seal drainage system (bottle) without routine suction.

Both groups received standard supportive care, including incentive spirometry, respiratory physiotherapy, encouragement, and oral acetaminophen (10 mg/kg every 6 h). All patients were advised to remain active. Supplemental oxygen via nasal cannula was administered if needed to maintain oxygen saturation >93 %. Intravenous ketorolac was available for breakthrough pain upon patient request and clinical assessment.

### Outcome measures

The primary outcomes were hospital stay length and time to normal activities, defined as patient-reported days from discharge to resuming daily routines/work. Other outcomes included daily pain assessment using a Visual Analog Scale (VAS) from 0 (no pain) to 10 (worst pain), and dyspnea assessed similarly from 0 (no breathlessness) to 10 (maximal breathlessness). Oxygen saturation (SpO2) was recorded. Ease of getting out of bed was gauged daily with a scale from 0 (unable/extremely difficult) to 10 (very easy). Analgesic requirements included total intravenous ketorolac dose (mg) and frequency. Treatment success was assessed based on complete lung re-expansion and the removal of the chest tube, complemented by clinical stability—defined *As spo*₂ >93 % on room air, no dyspnea, and symmetric breath sounds—and radiographic evidence of re-expansion on daily chest X-rays after 48 h. Discharge criteria were standardized and uniformly applied to both groups, including clinical stability, radiographic confirmation of re-expansion, and no air leaks. These criteria were prospectively established to minimize bias, even though the study was open-label. A persistent air leak indicated treatment failure after 3 days or a lack of full re-expansion, which necessitated consideration of VATS by the surgeon's decision. 30-day rehospitalization was defined as readmission for recurrent pneumothorax or complications within 30 days post-discharge. Complications included adverse events during hospitalization or within 30 days, such as subcutaneous emphysema, hemothorax/hematoma at the insertion site, and wound infection. Pneumothorax size was assessed via initial CT scans and daily chest radiographs (from day 3) by an experienced surgeon, based on the interpleural distance. The research team collected data prospectively using standardized forms, and follow-up occurred weekly for 30 days after discharge. Additionally, the time until chest drain removal was documented and analyzed, with removal decisions according to the same clinical and radiographic criteria used for discharge.

### Sample size

This study was conducted as an exploratory trial without a formal hypothesis-testing structure. The target sample size of 40 participants (20 per group) was selected based on feasibility and logistical considerations rather than a predefined power calculation. We recognize that this sample size was estimated without a specific minimal detectable difference defined, and therefore, statistical power to detect small differences may be limited. The intent was to gather preliminary data to inform future hypothesis-driven studies.

### Statistical analysis

Data were analyzed using SPSS version 26 (IBM Corp., Armonk, NY). Continuous variables were checked for normality using Kolmogorov-Smirnov and Shapiro-Wilk tests. Normally distributed continuous data were presented as mean ± standard deviation (SD) and compared using independent samples *t*-tests. Non-normally distributed continuous data were presented as median and interquartile range (IQR) and compared using the Mann-Whitney U test. Categorical data were presented as frequency (percentage) and compared using the Chi-square test or Fisher's exact test, as appropriate. Repeated measures (pain, dyspnea, ease of mobility over time) were assessed graphically and compared at specific time points using appropriate tests. A P-value < 0.05 was considered statistically significant. All randomized patients were analyzed based on the intention-to-treat principle. Patients needing VATS remained in their original groups, and their entire hospital stay, including post-surgical days, was used to determine the length of stay outcome.

## Results

### Participant flow and baseline characteristics

**Fig. 1:**
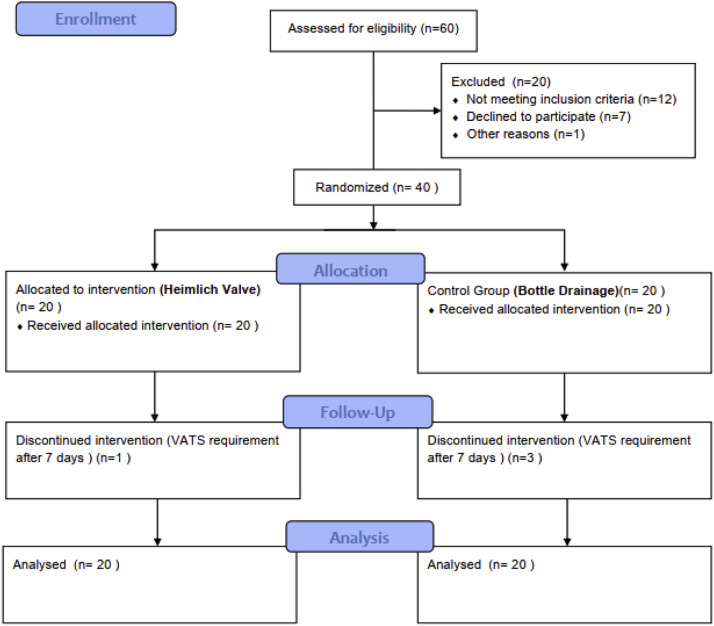
CONSORT flow diagram.

Forty eligible patients with PSP provided consent and were randomly divided into two groups: 20 to the Heimlich valve group and 20 to the chest tube with bottle group. Four patients—three from the chest bottle group and one from the Heimlich valve group—required VATS due to treatment failure; their outcomes were included in the analysis up to the point of surgery (Fig. 1).

Baseline demographic and clinical characteristics were well-balanced between the two groups (Table 1). The mean age was 31.1 ± 7.0 years overall, 80 % were male, and 50 % reported a smoking history. Comorbidities like hypertension, diabetes, and asthma were infrequent.

**Table 1:** Baseline demographic and clinical characteristics of study participants.

Characteristic	Heimlich Valve (n=20)	Bottle Drainage (n=20)	P-value	Overall (N=40)
Age (years), mean ± SD	30.5 ± 7.2	31.7 ± 6.9	0.59	31.1 ± 7.0
Male Sex, n ( %)	15 (75 %)	17 (85 %)	0.42	32 (80 %)
Height (cm), mean ± SD	172.4 ± 9.4	171.8 ± 6.2	0.82	172.1 ± 7.9
Weight (kg), mean ± SD	72.7 ± 11.9	70.1 ± 10.5	0.47	71.4 ± 11.2
BMI (kg/m ²), mean ± SD	24.3 ± 3.6	23.7 ± 3.2	0.49	24.0 ± 3.4
History of Smoking, n ( %)			0.92	
- Non-smoker	9 (45 %)	11 (55 %)		20 (50 %)
- Light (<5/day)	4 (20 %)	3 (15 %)		7 (17.5 %)
- Moderate (5–10/day)	2 (10 %)	2 (10 %)		4 (10 %)
- Heavy (>10/day)	5 (25 %)	4 (20 %)		9 (22.5 %)
History of Drug Use, n ( %)	6 (30 %)	5 (25 %)	0.72	11 (27.5 %)
History of Surgery, n ( %)	8 (40 %)	10 (50 %)	0.52	18 (45 %)
Hypertension, n ( %)	2 (10 %)	1 (5 %)	0.54	3 (7.5 %)
Diabetes Mellitus, n ( %)	3 (15 %)	2 (10 %)	0.63	5 (12.5 %)
Asthma, n ( %)	2 (10 %)	1 (5 %)	0.54	3 (7.5 %)
**Initial Presentation**				
Pneumothorax size ( %), mean ± SD	38.5 ± 10.9	40.8 ± 11.0	0.52	39.7 ± 10.9
Oxygen Saturation ( %), mean ± SD	91.9 ± 2.5	92.2 ± 2.5	0.88^a^	92.0 ± 2.5
Pain (VAS 0–10), median (IQR)	6.5 (6–7)	7.0 (6.5–7.5)	0.33^a^	7.0 (6–7.5)
Dyspnea (0–10), median (IQR)	4.5 (4–5.5)	5.0 (4–5.5)	0.82^a^	5.0 (4–5.5)
Ease of Getting out of Bed (0–10), median (IQR)	3.0 (2.5–3.5)	2.0 (1–2.5)	0.04^a^	2.5 (1.5–3)

Abbreviations: SD, standard deviation; BMI, body mass index; VAS, Visual Analog Scale; IQR, Interquartile Range.

P-values from independent *t*-tests for continuous variables (age, height, weight, BMI, pneumothorax size) or Chi-square/Fisher's exact test for categorical variables unless otherwise indicated.

a =P-value from the Mann-Whitney U test due to non-normal distribution or ordinal scale.

## Clinical outcomes

### Length of stay and recovery

The mean length of hospital stay was numerically shorter in the Heimlich valve group (5.60 ± 3.01 days) compared to the bottle drainage group (7.25 ± 4.57 days), but this difference did not reach statistical significance (P=0.081). However, the mean time reported to return to normal daily activities was significantly shorter for patients in the Heimlich valve group (7.10 ± 5.44 days) compared to the bottle drainage group (10.15 ± 8.00 days, P=0.014).

### Other outcomes

**Fig. 2:**
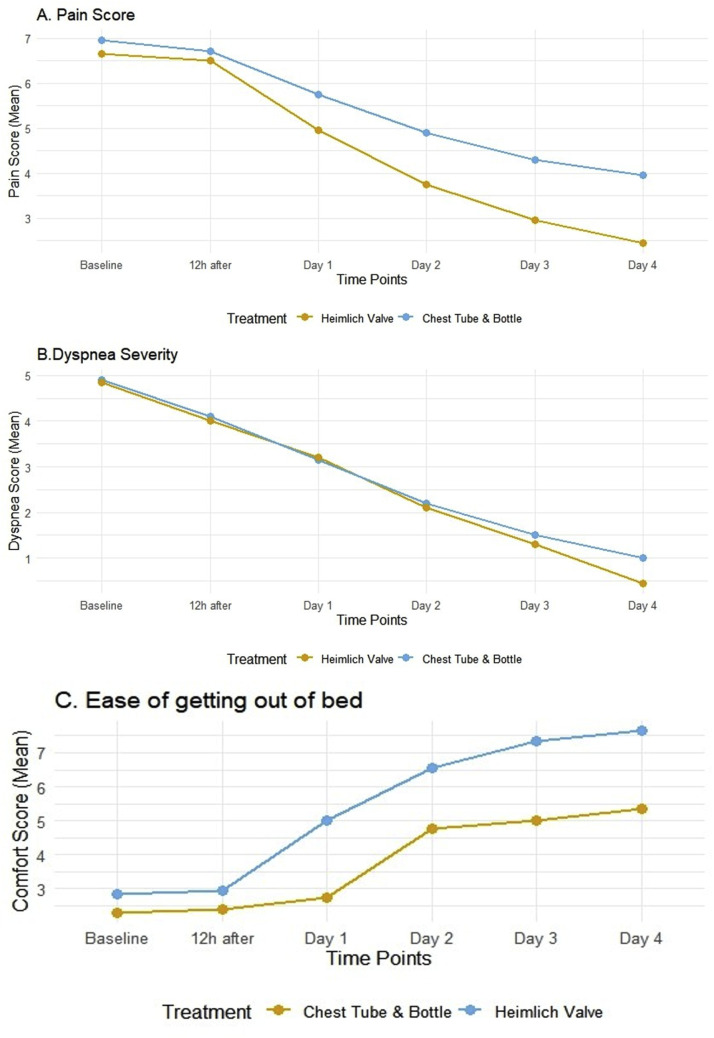
(A) Pain scores, (B) Dyspnea score, (C) ease of getting out of bed with the Heimlich valve compared to the bottle drainage group.

Although baseline pain scores were comparable, daily assessments showed the Heimlich valve group reported significantly lower pain scores compared to the bottle drainage group on day 1 (mean VAS 4.95 vs. 5.75, P=0.008), day 2 (3.75 vs. 4.90, P<0.001), day 3 (2.95 vs. 4.30, P<0.001), and day 4 (2.45 vs. 3.95, P<0.001) (Fig. 2 A, Table 2). Thus, patients in the Heimlich group required fewer ketorolac doses, averaging 3.20 ± 0.95 doses, compared to 4.90 ± 1.37 doses in the bottle group (P<0.001). The mean total ketorolac dose was also significantly lower for the Heimlich group (98 ± 26 mg vs. 147 ± 41 mg, P<0.001). The mean time to chest drain removal was significantly shorter in the Heimlich valve group (3.8 ± 1.1 days) compared to the bottle drainage group (5.0 ± 1.3 days), P = 0.019. Dyspnea scores showed a progressive decrease in both groups (Fig. 2 B, Table 2). By day 4, the Heimlich group had a mean dyspnea score of 0.45 ± 0.68, trending lower than the bottle group's score of 1.00 ± 1.16, although this difference was not statistically significant (P=0.078). Oxygen saturation levels improved similarly in both groups over time (Table 2). The Heimlich valve group also reported significantly greater ease of getting out of bed at all time points assessed from baseline through day 4 compared to the bottle group (P<0.05 for all comparisons) (Fig. 2 C, Table 2). The mean percentage of pneumothorax decreased in both groups. On day 3, the mean size was 20.5 ± 8.7 % in the Heimlich group compared to 24.8 ± 10.8 % in the bottle group (P=0.179). By day 4, it was 9.8 ± 9.9 % versus 16.3 ± 12.6 %, respectively (P=0.077), indicating a trend towards faster resolution in the Heimlich group (Table 2). Treatment failure that required VATS occurred in 1 patient (5 %) from the Heimlich valve group and 3 patients (15 %) from the bottle drainage group; however, this difference was not statistically significant (P=0.29). Additionally, one patient (5 %) from each group was rehospitalized within 30 days due to recurrence (P=1.00).

**Table 2:** Comparison of outcomes over time between treatment groups (mean ± SD or median (IQR)).

Outcome Measure	Time Point	Heimlich Valve (n=20)	Bottle Drainage (n=20)	P-value
**Pain (VAS 0–10)**	Baseline	6.65 ± 0.74	6.95 ± 0.88	0.254^b^
	12 h	6.50 ± 0.94	6.70 ± 1.08	0.537^b^
	Day 1	4.95 ± 0.82	5.75 ± 0.96	**0.008**^b^
	Day 2	3.75 ± 0.78	4.90 ± 0.85	**<0.001**^b^
	Day 3	2.95 ± 0.68	4.30 ± 1.03	**<0.001**^b^
	Day 4	2.45 ± 0.51	3.95 ± 0.88	**<0.001**^b^
**Dyspnea (0–10)**	Baseline	4.85 ± 1.18	4.90 ± 1.20	0.896^b^
	12 h	4.00 ± 1.02	4.10 ± 1.25	0.784^b^
	Day 1	3.20 ± 0.89	3.15 ± 1.22	0.884b
	Day 2	2.10 ± 1.16	2.20 ± 1.28	0.798^b^
	Day 3	1.30 ± 1.21	1.50 ± 1.23	0.609^b^
	Day 4	0.45 ± 0.68	1.00 ± 1.16	0.078^b^
**Ease of Getting out of Bed (0–10)**	Baseline	2.85 ± 0.74	2.30 ± 0.80	**0.030**^b^
	12 h	2.95 ± 0.68	2.40 ± 0.88	**0.034**^b^
	Day 1	5.00 ± 0.64	3.75 ± 0.71	**<0.001**^b^
	Day 2	6.55 ± 0.68	4.75 ± 0.91	**<0.001**^b^
	Day 3	7.35 ± 0.58	5.00 ± 0.91	**<0.001**^b^
	Day 4	7.65 ± 0.74	5.35 ± 0.74	**<0.001**^b^
**Oxygen Saturation ( %)**	Baseline	91.9 ± 2.5	92.2 ± 2.5	0.751^b^
	12 h	93.4 ± 1.3	93.6 ± 1.4	0.727^b^
	Day 1	94.9 ± 0.8	94.9 ± 1.0	0.865^b^
	Day 2	95.5 ± 0.8	95.6 ± 1.2	0.645^b^
	Day 3	95.5 ± 0.9	95.5 ± 1.3	1.000^b^
	Day 4	95.9 ± 0.8	95.9 ± 1.0	0.730^b^
**Pneumothorax Size ( %)**	Baseline	38.5 ± 10.9	40.8 ± 11.0	0.520^c^
	Day 3	20.5 ± 8.7	24.8 ± 10.8	0.179^c^
	Day 4	9.8 ± 9.9	16.3 ± 12.6	0.077^c^

Abbreviations: VAS, Visual Analog Scale; SD, standard deviation.

b=P-value likely derived from Mann-Whitney U test based on thesis methods description of non-normality for these scales/measures, though means ± SD were reported.

c=P-value from independent *t*-test (Pneumothorax size reported as normal distribution in thesis).

Bold P-values indicate statistical significance (P<0.05).

### Adverse events

No major complications like hemothorax occurred. Minor complications were infrequent and similar between groups. Subcutaneous emphysema was observed in 2 patients (10 %) in the Heimlich group and 3 patients (15 %) in the bottle group (P=0.63). Insertion site wound infection occurred in 1 patient (5 %) in the bottle group and none in the Heimlich group (P=0.31). No insertion site hematomas were reported.

## 4 Discussion

This randomized controlled trial compared the use of a Heimlich one-way valve versus conventional underwater seal bottle drainage, connected to a standard 28 Fr chest tube, for the initial management of primary spontaneous pneumothorax in young adults. The main findings indicate that the Heimlich valve group experienced less pain, required less analgesia, reported greater ease of mobilization, and returned to normal activities significantly faster than the bottle drainage group. While not statistically significant, trends towards faster pneumothorax resolution and shorter hospital stays were also observed in the Heimlich valve group. Importantly, the safety profiles appeared similar, with low and comparable rates of minor complications and treatment failure.

The observed reduction in pain with the Heimlich valve is a clinically relevant finding. This may be partly attributed to the increased mobility facilitated by the smaller, lighter valve system compared to the cumbersome bottle drainage [14,15]. Reduced pain likely contributed to the lower requirement for ketorolac analgesia. This contrasts with some studies that found no difference in pain scores between ambulatory management (which often uses valves) and standard care [11,16]. However, study populations (PSP vs. SSP) and specific devices varied. Our findings align more closely with those of Rasihashemi et al.[12], who reported less procedural and removal pain and lower analgesic use with pigtail catheters (often used with valves) compared to larger chest tubes. However, our study used a chest tube of the same size in both arms, isolating the effect of the drainage system itself.

The significantly faster return to normal activities in the Heimlich group is a key advantage, potentially translating to reduced socioeconomic impact. This is likely multifactorial, related to reduced pain, improved mobility during the hospital stay, and possibly the non-significant trend towards shorter hospitalization. Hallifax et al. [11] also found that ambulatory management significantly reduced hospital stay duration in PSP patients. While our study did not show a statistically significant reduction in length of stay (P=0.081), the numerical difference of approximately 1.6 days warrants further investigation in larger trials, as even modest reductions can impact healthcare costs and resource utilization[17].

The trends towards faster pneumothorax resolution (P=0.077 on Day 4) and lower rates of treatment failure requiring VATS (5 % vs. 15 %, P=0.29) in the Heimlich group are encouraging but require cautious interpretation due to the lack of statistical significance, likely related to the small sample size. Some literature suggests similar overall success rates between different drainage methods [12,18,19], while others report variations [16]. The mechanisms by which a Heimlich valve might promote faster resolution compared to a simple underwater seal are unclear but could relate to more consistent negative pressure generation during movement or coughing, although this is speculative.

Our study confirms the safety of the Heimlich valve in this setting, with low complication rates similar to the standard bottle drainage group, consistent with previous reports [7,17]. The rates of subcutaneous emphysema and wound infection were minimal.

This study has several limitations due to its nature. Firstly, it was a single-center trial with a relatively small sample size (n=40), limiting the statistical power to detect minor differences, particularly for binary outcomes like treatment failure or complications. Secondly, the open-label design introduces potential for performance and detection bias, although objective measures like length of stay and radiographic resolution are less susceptible. Thirdly, patient-reported outcomes like pain, dyspnea, and ease of mobility rely on subjective assessment, though standardized scales were used. Finally, estimating pneumothorax size from radiographs can be imprecise [20].

The findings are likely generalizable to young adults (18–40 years) presenting with a first episode of symptomatic PSP requiring chest tube drainage in similar healthcare settings. Applicability to older patients, those with secondary spontaneous pneumothorax, or recurrent PSP may differ. Although standardized criteria were applied for discharge and drain removal, the open-label design may lead to detection bias, especially for subjective outcomes. This underscores the importance of future studies involving blinded or objective outcome assessors.

## Conclusion

Compared to conventional chest tube drainage with an underwater sealed bottle, using a Heimlich one-way valve for managing primary spontaneous pneumothorax significantly reduced pain, analgesic requirements, and time to return to normal activities, while enhancing patient mobility. Trends suggested faster pneumothorax resolution and potentially shorter hospital stays. The Heimlich valve appears to be a safe and effective alternative that offers tangible benefits in patient comfort and functional recovery. Larger, multi-center trials are warranted to confirm these findings, particularly regarding length of stay and treatment failure rates.

## Funding

The Vice-Chancellor for Research and Technology of Alborz University of Medical Sciences, Karaj, Iran, financially supported this study.

## Duplicate submission and fraudulent publication

This manuscript has not been previously published and is not under consideration elsewhere.

All data presented are accurate and original; no part of this work has been falsified or fabricated.

## Use of artificial intelligence (AI) in manuscript preparation

Generative AI tools (e.g., ChatGPT) were used solely as an editorial assistant to refine language and style. No data or research findings were generated or manipulated by AI. All authors have reviewed and take full responsibility for the integrity of the content.

## Statement of informed consent

All individual participants in this study provided informed consent, and additional consent was obtained as required for any identifying information.

## Statement of human rights

The ethics committee of Alborz University of Medical Sciences, Alborz, Karaj, Iran, approved this study. (Code: IR.ABZUMS.REC.1401.272). All procedures followed the ethical guidelines of the Declaration of Helsinki and COPE.

## CRediT authorship contribution statement

**Mohammad Hadi Bahri:** Validation, Supervision, Software, Resources, Project administration, Methodology, Investigation, Funding acquisition, Formal analysis, Data curation, Conceptualization. **Seyed Mohammad Naghibalghora:** Writing – review & editing, Writing – original draft, Software, Resources, Project administration, Methodology, Investigation, Formal analysis, Data curation, Conceptualization. **Mojtaba Ahmadinejad:** Supervision, Project administration, Methodology, Investigation, Conceptualization. **Kourosh Kabir:** Supervision, Software, Methodology, Formal analysis, Data curation, Conceptualization. **Nazanin Khezri:** Writing – original draft, Data curation, Conceptualization.

## Declaration of competing interest

The authors declare the following financial interests/personal relationships which may be considered as potential competing interests: Seyed Mohammad Naghibalghora reports financial support and equipment, drugs, or supplies were provided by Alborz University of Medical Sciences, Karaj, Iran. Seyed Mohammad Naghibalghora reports a relationship with Alborz University of Medical Sciences that includes: funding grants. The other authors declare that they have no known competing financial interests or personal relationships that could have appeared to influence the work reported in this paper.

## Data Availability

The data supporting this study's findings are available from the corresponding author upon reasonable request.
